# Abstract of the Proceedings of the Ninth Annual Session of the American Medical Association, Held in Detroit, May 6, 7, 8, and 9, 1856

**Published:** 1856-07

**Authors:** 


From the Medical Counselor.

### *Abstract of the Proceedings of the Ninth Annual Session of the American Medical Association, held in Detroit, May* 6, 7, 8 *and* 9, 1856.

*Prepared by the Editor.*

Dr. Wister, of Pa., from the committee on Publication, made the annual report. It states that the first copies of the transactions of the last session of the Association were issued on the 10th of November, 1855; that 1,100 copies were printed; that the aggregate expense of printing, illustrating, and binding was $1,922.70; that the distribution of the volume was effected, in every possible instance, by express; that Drs. C.

Hooker of Ct., Alden March of Albany, J. L. Atlee of Pa., W. Brodie of Mich., C. B. Gibson of Richmond, E. L. Beadle of N. Y., H. W. Dessaussure of S. C., C. A. Pope of Mo., D. H. Storer of Mass., T. G. Richardson of Ky., J. Moran of R. I., T. Miller of D. C., F. E. B. Hintze of Md., L. P. Bush of Del., Z. Pitcher of Mich., and J. B. Lindsley of Tenn., have rendered essential service to the Association—some in procuring subscriptions to the volume, and all by cordial coöperation in its distribution; that it is important to secure efficient coöperation in every State by the appointment of gentlemen whose duty it shall be to aid in procuring subscriptions for and circulating the transactions; that Connecticut is especially to be commended for her services in this particular; that not a little embarrassment was experienced by the committee in restoring to the list of permanent members the names of those who had been left off by order of the Association for non-payment of assessments; that they had endeavored, however, by careful comparison of the various lists, to supply all omissions; that the committee had been reluctantly obliged to omit from the transactions two valuable reports on epidemic diseases by Dr. L. H. Anderson, of Alabama, and Dr. E. D. Fener, of New Orleans; but, as they had not been presented to the Association, and acted on by that body, there was no other alternative; that the following resolution, passed at the last session, should be strictly enforced:

*Resolved,* That, hereafter, beginning with the session of 1856, no report, or other paper shall be entitled to publication in the volume for the year in which it shall be presented to the Association, unless it be placed in the hands of the committee of Publication on or before June 1st.

The report further states that the number of volumes of transactions now remaining on hand is as follows: of Vol. I. 41, of Vol. II. 9, of Vol. III. 32, of Vol. IV. 7, of Vol. V. 316, of Vol. VI. 66, of Vol. VII 120, of Vol. VIII. 351; that some of the leading journals abroad have expressed a strong desire to complete their sets, and it rests with the Association to determine whether the missing numbers shall be supplied; that, as only seven complete sets of the transactions are now in the possession of the Association, the committee recommend that no copy of either of the eight volumes which is necessary to the complete sets now remaining shall be disposed of separately, or with any number of volumes short of a complete set.

Dr. Atlee, of Pa., made some remarks upon the report, in the course of which he stated that the Smithsonian Institution had been offered as a permanent place of session for the Association. He concluded by moving that the committee on Publication preserve five complete sets of the proceedings. Carried.

Dr. Wood, of Philadelphia, moved to refer the nomination of standing committees to the committee on Nominations. Carried.

The same gentleman made a request, in behalf of Dr. Hamilton, that the committee of which Dr. H. is chairman may be continued for another year, it not being prepared to report at present. Granted.

Dr. Breckenbridge, of Ky., stated that the committee on Medical Literature was ready to report.

The President suggested that the reading of the report follow that of the report of Dr. Gross, which had been made the special order for Wednesday, at 10 A. M.

Dr. Palmer, of Chicago, stated that the committee on Plan of Organization for State and County Medical Societies was ready to report.

Dr. Atlee, of Pa., moved to refer the prize essay of Dr. Hartshorn on Arterial Circulation, and the report of Dr. Blatchford on Hydrophobia, to the committee on Publication. Carried.

Dr. Wister, of Pa., the Treasurer, read his annual report. It recommends that the Treasurer be requested, at an early date after the adjournment of the present meeting, to address a circular to each permanent member, announcing the abrogation of the resolution of 1854—making a yearly subscription to the transactions obligatory—and the consequent restoration to membership of all those dropped from the published list of that year, advertising, also, the practicability of procuring back numbers of the transactions, with information as to the cost at which the series of volumes may be rendered complete, or an entire set furnished by the Association.

The account of the Treasurer with the Association is as follows:

DR.

| | |
|---|---|
| To cash paid to Dr. John L. Atlee, of committee on Washington Monument Stone, | $498 70 |
| To cash paid C. B. Norton, for porterage and packing Vol. VII, in New York, | 8 00 |
| To cash paid J. D. Trask, for Prize Essay, | 100 00 |
| To cash paid for postage of Secretary, | 2 50 |
| To cash paid D. C. Baxter, for engravings of Vol. VIII, | 72 75 |
| To cash paid for postage of Chairman of Publication Committee, | 4 09 |
| To cash paid Thos. Sinclair & Co., for lithographs for Vol. VIII, | 101 20 |
| To cash paid T. R. & P. G. Collins for printing and binding 1,100 copies of Vol. VIII, | 1,748 75 |
| To cash paid T. R. & P. G. Collins for binding 25 copies Vol. VI, and printing notices, | 4 52 |
| To cash paid H. Barnes for distribution of Vol. VIII, and services as clerk, | 50 00 |
| To cash paid T. R. & P. G. Collins for printing notices, | 1 25 |
| To cash paid Blanchard & Lea for freight, porterage, boxes, etc., for Vol. VIII, | 34 99 |
| To cash paid for postage, envelopes, and stationery of Treasurer | 6 99 |
| To balance, | 950 52 |
| | $3,584 26 |

CR.

| | |
|---|---|
| By cash received from Dr. Isaac Wood, being the balance in the Treasury April 30th, 1855,........................................ | $1,015 26 |
| By cash received from Dr. Isaac Wood, being the balance in the Treasury of prize essay fund, April 30th, 1855,......... | 100 00 |
| By cash received from assessment and the sale of transactions, | 2,150 50 |
| By cash received from Dr. E. L. Beadle for the sale of transactions,........................................................................ | 12 00 |
| By cash received from Dr. Wm. Brodie for do,.................... | 12 00 |
| By cash received from Dr. A. March for do........ .............. | 24 00 |
| By cash received from Messrs. Blanchard & Lea for do....... | 102 50 |
| By cash received from Dr. Chas. Hooker for do.................. | 168 00 |
| | $3,584 26 |

The correctness of this account is certified to by the proper committee.

The report was accepted, and referred to the committee on Publication.

The President read a communication from Dr. Stewart, chairman of the committee appointed last year to consider the subject of extending the lectures of each chair in medical schools over a period of two years, stating that the views of medical institutions had as yet been imperfectly ascertained, and asking a continuance of the committee. Granted.

The President read an invitation to the Association to attend the session of the American Association for the advancement of Science, at Albany, in August next,—at which time, also, the Dudley Observatory will be inaugurated, and an address delivered by Hon. Edward Everett. The invitation was accepted.

SECOND DAY.

The Association was called to order by the President, at nine o'clock.

The minutes were read, corrected and approved.

The Secretary announced that he had received the following resolution adopted at the last meeting of the New York State Medical Society:

*Resolved*, That the members of the American Medical Association be invited to attend the semi-centennial celebration of this society, which will occur on the first Tuesday of February, 1857.

The invitation was accepted.

The Secretary read the following communication, dated April 7, 1856, from the Secretary of the Ohio State Medical Society:

Sir:—At the annual meeting of this society, held in June last, at Zanesville, Ohio, the following resolutions were adopted, and I was directed to furnish you with a copy of the same:

*Resolved*, That the resolution offered by Dr. Grant, (a member of this society, but not at this or at that time a practitioner of medicine, but a lawyer,) at the last session of this society, viz: "That it is not derogatory to medical dignity, or inconsistent with medical honor, for medical gentlemen to take out a patent right for surgical or mechanical instruments," was offered at a time when many members had left for their homes, and is not, therefore, the sense of the society.

*Resolved*, That the said resolution is in direct opposition to the code of medical ethics adopted by this society; and, therefore, be it further

*Resolved*, That said resolution, offered by Dr. Grant, and adopted by the society, be and is hereby, rescinded.

The communication was ordered to be placed upon the minutes.

The Secretary read a communication from Dr. Hamilton, of Buffalo, N. Y., transmitting the second part of a report upon Deformities after Fracture and Dislocations, and asking for a correction of the minutes of last session in regard thereto. Dr. H. also asked that he be permitted to incorporate, in a volume upon the subject he is preparing for publication, that portion of the report already published by the Association.

On motion of Dr. Brodie, of Mich., the minutes were amended.

Dr. Atlee, of Pa., offered a resolution that the request of Dr. H., in regard to the publication of the report, be granted.

Dr. Lindsley, of Tenn., opposed the resolution. A similar request was denied at the session of the Association held at St. Louis.

Dr. Palmer, of Ill., moved to refer the matter to a special committee. Carried.

The President appointed as such committee, Drs. Palmer, of Ill., Atlee, of Pa., and Hills, of Ohio.

Dr. Gunn, of Mich., moved that those gentlemen from Canada, who are here by general invitation, be admitted in a body, and be requested to take seats on the platform during this morning's session. Carried.

The following gentlemen complied with the invitation:

Dr. E. M. Hodder, F. R. S. Eng., Prof. of Midwifery and Diseases of Children, Trinity College, Toronto.

Dr. J. H. Richardson, M. R. C. S. Eng., Examiner in Anatomy, University of Toronto.

Dr. Norman Bethune, M. R. C. S. Eng., Prof. of Anatomy, Trinity College, Toronto.

Dr. Worthy Haswell, M. R. C. of Surgery, Eng.

Dr. A. K. Dewson, College Physicians and Surgeons, N. Y., Licentiate of Province of the Canadas.

Dr. John Tarquand, Woodstock, C. W.

Dr. Geo. Coatsworth, Medical Department University of Buffalo, Licentiate of Province of the Canadas.

In receiving them upon the platform, the President, Dr. Pitcher, said he was happy to be the instrument of celebrating the nuptials by which we effect a scientific re-union of the two members of the Anglo-Saxon race, which have so long been separated by the political relations having their origin in the separation of the American colonies from the English crown.

Dr. Hodder, in behalf of his Canadian brethren, thanked the Association for the courtesy and kindness extended to them.

Dr. Sutton, of Ky., offered a resolution that 1,000 copies of the address of the late President, Dr. Wood, be published. Adopted.

On motion of Dr. J. B. Lindsley, of Tenn.,

*Resolved*, That a committee of three be appointed by the Chair, to prepare a suitable minute in reference to the death of our late Secretary, Dr. P. C. Gooch, of Richmond, Va., who fell a martyr while contending with the pestilence in Norfolk, in 1855.

The President appointed as such committee, Drs. Lindsley, of Tenn., Thomson, of Del., and Mendenhall, of Ohio.

Dr. Gross, of Ky., from committee appointed the day previous, reported the following preamble and resolutions relative to the death of Dr. J. C. Warren, of Boston:

*Whereas*, It has pleased Almighty God to remove from the scene of his earthly labors our late fellow-member, Dr. John C. Warren, of Boston, formerly President of this Association, and for many years Professor of Anatomy and Surgery in Harvard University;

*And whereas*, It is just and proper that, when a great and good man dies, his memory should be cherished by his fellow-citizens, and transmitted unimpaired to posterity for the encouragement of future ages; it is therefore,

*Resolved*, That this Association has learned with profound regret the news of an event which has deprived the American Medical profession of one of its oldest, most useful, and most illustrious members—American surgery one of its greatest ornaments—science one of its best friends—and humanity one of its noblest benefactors.

*Resolved*, That the life of Dr. John C. Warren affords an example of a man who, notwithstanding the possession of ample riches, devoted himself, heart and soul, for upwards of half a century, to the cultivation and advancement of his profession, and to the good of the human race.

*Resolved*, That this Association deeply sympathizes with the family of Dr. Warren in their bereavement, and that the Secretary be requested to transmit to them a copy of these proceedings.

The preamble and resolutions were adopted and referred to the committee on Publication.

Dr. Gross, of Ky., read a report on "The Causes which Impede the Progress of American Medical Literature." In conclusion, he submitted the following resolutions:

*Resolved*, That this Association earnestly and respectfully recommends: 1st. The universal adoption, whenever practicable, by our schools, of American works, as text-books for their pupils. 2d. The discontinuance of the practice of editing foreign writings. 3d. A more independent course of the medical periodical press towards foreign productions, and a more liberal one towards American; and, 4th. A better and more efficient employment of the facts which are continually furnished by our public institutions, for the elucidation of the nature of diseases and accidents, and, indirectly, for the formation of an original, a vigorous, and an independent national medical literature.

*Resolved*, That we venerate the writings of the great medical men, past and present, of our country, and that we consider them as an important element of our national medical literature.

*Resolved*, That we shall always hail with pleasure any useful or valuable work emanating from the European press, and that we shall always extend to them a cordial welcome, as books of reference, to acquaint us with the progress of legitimate medicine abroad, and to enlighten us in regard to any new facts of which they may be the repositories.

Dr. Phelps, of New York, moved that the report and resolutions, as a whole, be adopted.

At the suggestion of a member, the question was divided. The report was adopted.

Upon the reading of the first resolution, a member proposed to substitute "just" for "liberal" in line 8. Dr. Gross accepted the amendment.

Dr. Palmer, of Ill., wished to understand the meaning of the word "practicable," as employed in the resolution, (line 3.) If it meant that an inferior work by an American author was to be used in our medical schools, in preference to a superior one, treating on the same subject, by a foreign author, he was decidedly opposed to the resolution. If, when the American work is equal or superior to the foreign one, it is to be used, then he had no objection. He alluded to works by eminent English and French authors.

Dr. Gross explained. One of the works alluded to by Dr. P. must of necessity be used in our medical institutions of learning, as there is no work by an American author on the same subject. Foreign works should be used as books of reference, and American books, "when practicable," as text-books.

Dr. Yandee, of Ky., moved that the resolutions be made the special order for Thursday morning. Lost.

Dr. Cobb, of New York, was opposed to the resolutions. If adopted and sent out to the world, they savor too much of Know-Nothingism to make them palatable. [Sensation.]

Dr. Leidy, of Pa., was in favor of leaving to teachers of medicine the selection of their own text-books.

Dr. Davis understood there was another report touching upon the subject—that upon "American Medical Literature," by Dr. Breckenridge, of Ky. He moved to lay the resolutions upon the table until that report was read. Carried.

The Secretary read a communication from Dr. P. A. Jewett, of Conn., chairman of the committee to Procure Memoirs of the Eminent and Worthy Dead. Referred to committee on Nominations.

Dr. Breckenridge, of Ky., read a report upon American Medical Literature.

On motion of Dr. Hooker, it was accepted and referred to the committee on Publication.

The Association then adjourned.

THIRD DAY—MORNING SESSION.

The Association was called to order, Dr. Z. Pitcher in the chair.

The minutes of previous meetings were read, and Dr. Hooper moved to amend the minutes by striking out so much as relates to the resolutions of Dr. Gross. Carried.

Dr. Allen, of R. I., moved the following:

*Resolved*, That the President shall be authorized annually to appoint delegates to represent this Association, at the meetings of the British Association, the American Medical Association at Paris, and such other scientific bodies in Europe as may be affiliated with us. Adopted.

Dr. L. D. Lutes, of N. Y., offered the following:

*Resolved*, That it is derogatory to the dignity of the Medical Profession to notice the works of irregular practitioners in Medical periodicals. Carried.

Dr. Clendennin offered the following:

*Resolved*, That a committee of one be appointed for a period of three years, with instructions to report the progress at each annual meeting of this Society, to investigate the Etiology and Pathology of Epidemic cholera, and that said committee be allowed to add any other member to the same, whom they may think necessary, to further the objects of their appointment. Referred to committee on Nominations.

Dr. Bissell, of Utica, N. Y., offered the following:

*Resolved*, That the Association has learned with deep regret the death of one of its members, Dr. Theodore Romeyn Beck, of Albany, N. Y., whose whole life has been devoted to the attainment and promotion of medicine and general science, and that we do hereby express our high appreciation of the excellencies of his character, distinguished by its simplicity, integrity and firmness of purpose, and by the extent and variety of his acquirements in medical as well as in almost every other department of science.

*Rsolved*, That the resolution be referred to a committee to procure memorials of the eminent and worthy dead, and that they be requested to procure a suitable memoir of the late Dr. Beck, to be published in the Transactions of the Association. Referred to Nominating Committee.

Dr. Mendenhall moved to strike from the list of perpetual members the name of Dr. Cleveland, Eclectic physician, of Cincinnati. Carried.

Dr. Atlee moved to strike from the same list, the name of James R. McClintock. Carried.

Dr. Bloodgood of Illinois, offered the following:

*Resolved*, That the Constitution of this Association be so amended as that hereafter the President shall be elected by ballot, and shall not be a resident of the State in which he is elected. Tabled.

Dr. James L. Phelps of N. Y., offered the following:

*Whereas*, It has pleased an all-wise but inscrutable Providence to visit the city of Norfolk, Va., and vicinity, with a desolating pestilence, equal or surpassing any recorded in ancient or modern times, and by which, in a few weeks, forty physicians, either residents or from abroad, who had promptly rushed to the rescue, among the number of whom was our late Secretary and associate, Dr. Gouch of Richmond; Therefore,

*Resolved*, That such an instance of signal and unflinching devotion to the cause of science and of humanity demands at the hands of their National Association, a passing expression of their high admiration of this, another memorable instance of the unparalleled sacrifices of the profession to the interests of the healing art, and of the race.

*Resolved*, That the sense of this Association be expressed by a rising vote, and that this minute be incorporated into our transactions. Carried.

The reports of special committees then being in order, the resolutions appended to the report of Dr. Gross were taken up.

Dr. Davis, of Illinois, moved to amend the records by striking out the adoption of the report of Dr. Gross, and to make them read "accepted and referred for publication."

Adopted and resolutions reëmbraced in report.

The Secretary presented communications from Dr. L. H. Steiner, of Baltimore, of committee to report on Strychnia, its chemical and toxicological parties, submitting that paper and expressing his regret at not being able to be present. Accepted and report referred for publication.

Dr. Palmer, of Ill., of special committee to report on request of Dr. Hamilton, offered the following:

*Resolved*, That leave be granted to Dr. F. H. Hamilton, of Buffalo, to make use of the material of his report on "deformities after fractures," which is in course of presentation to this Association, in his anticipated work on "Fractures and Dislocations," provided that book is not issued until after the report has appeared in the Transactions of this Association. Adopted.

Dr. Palmer, of Illinois, of committee to report on the Plans of Organization of State and County Medical Societies, presented his report, which was accepted and referred for publication.

Dr. Davis of Ill., of committee to report on the Compositions and Changes of the Milk of the Human Female, produced by Menstruation and Pregnancy, produced his report, which was accepted and referred for publication.

The Secretary read a communication from C. Q. Chandler, Mo., of committee to report upon Malignant Periodic Fevers, stating that he had not been able to furnish a paper upon that subject, but offered in its place one upon the Sulphate of Cinchona. Accepted, and the paper referred to a special committee.

Dr. H. A. Johnson, of Ill., from committee to report on the Excretions as an Index to the Organic Changes going on in the System, read his report, which was referred for publication.

Several other special committees were called but their reports had been deferred, as they were appointed for three years.

The report of Dr. J. A. Newman, of Buffalo, on the sanitary policies of cities, was referred for publication, when the Association adjourned to 2 o'clock.

AFTERNOON SESSION.

The President called the Association to order at 2 o'clock.

Dr. Frost, of S. C., before the resumption of the regular order, asked leave to offer the following:

*Resolved*, That the thanks of this Association are due to the retiring officers, for the zealous and efficient manner in which their duties have been performed; to our late President for the courteousness and ability with which he has presided over our deliberations; to all the officers for their attention to the laborious duties of their stations, not excepting our committee on Publication, to whom we must feel indebted for the satisfactory form in which the volume of Transactions appears. Carried.

The regular order was then called, and Dr. A. J. Green, of Me., reported on the best treatment of Cholera Infantum.

His report was accepted, and referred to the committee on Publication.

Dr. Horace Green, of N. Y., reported on the application of Nitrate of Silver to the throat. Approved and referred to the committee on Publication.

Dr. J. B. Flint, of Ky., reported on the best mode of rendering the medical patronage of the National Government tributary to the honor and improvement of the profession. Accepted, and referred for publication.

The committee on Nominations, reported the following committees for the ensuing year:

Dr. E. K. Peaslee, of Brunswick, Me, on Inflammation, its pathology and relation to the recuperative process.

J. C. Hutchinson, of Brooklyn, N. Y., and Charles E. Isaacs, of N. Y., on the anatomy and histology of the Cervix Uteri.

J. T. Bradford, of Augusta, Ky., on the treatment of Cholera.

M. Stevenson, of N. Y., on the treatment best adapted to each variety of Cataract, with the method of operation, place of election, time, age, etc.

J. W. Corson, of N. Y., on the causes of the impulse of the heart, and the agencies which influence it under health and disease.

D. M. Ruse, of N. Y., on the causes of infant mortality in large cities, and the sources of its increase, and the means of its diminution.

J. F. Jenkins, of Yonkers, N. Y., on spontaneous Umbilical Hemorrhage of the newly-born.

H. Carpenter, of Pa., on the use of instruments in obstetric practice.

A. J. Summer, of D. C., on the means to be adopted to remedy the evils in the present mode of holding coroner's inquests.

J. M. Simms, of N. Y., on the treatment of the results of obstructed labor.

J. B. Flint, of Ky., on the true position and value of operative Surgery as a therapeutic agent.

G. V. Dorsey, of Ohio, on the causes and cure of Indigestion, especially in relation to the therapeutic indications to be derived from the chemical composition of the deposits in the urine.

C. B. Coventry, of N. Y., on the Medical Jurisprudence of Insanity, and the testimony of skilled witnesses in Courts of Justice.

J. Leidy, of Pa., on Human, Animal, and Vegetable Parasites.

M. D. Darnall, of Ind., on the value of a strict attention to position in the treatment of diseases of the abdomen.

G. Sutton, of Ind., on Milk Sickness.

C. J. Pease, of Wis., on the blending and conversion of the types of Fever.

B. S. Woodworth, of Ind., on the best substitute for Cinchona and its Preparations in the treatment of Intermittent Fevers and malarious diseases.

H. F. Campbell, of Ga., on the nervous condition in febrile diseases.

J. Morse, of N. Y., on the diseases incident to Europeans from temperate climates, in their transition through Central America.

J. Neill, of Pa., on the laws governing the deposits of bone.

J. W. Green, of N. Y., on the intimate effects of certain toxicological agents in the mineral tissues and fluids

Geo. Suckling, of the U. S. Army, on the medical topography and Farina of Washington territory.

James Cooper, of N. J., on the Flora of Washington and Oregon territories.

C. E. Jones, of N. Y., on the intimate structure and pathology of the kidneys.

T. W. Gordon, of Ohio, on the etiology and pathology of epidemic Cholera, to be continued three years, and with power to add any other member to his committee.

D. D. Thompson, of Ky., on the remedial effects of Chloroform. Adopted.

STANDING COMMITTEES.

*Committee on Publications.*—F. G. Smith, Pa.; Caspar Wister, Pa.; S. L. Hollingsworth, Pa.; S. Lewis, Pa.; H. F. Askew, Del.; W. Brodie, Mich.; R. C. Foster, Tenn.

*On Prize Essays*—W. K. Bowling, E. B. Haskins, T. Lipscomb, A. H. Buchanan, B. W. Avent, W. A. Cheatham, and P. F. Eve, Tenn.

*On Arrangements*—C. K. Winston, Ira Conwell, W. D. Haggart, J. L. C. Johnson, V. A. Ramsey, G. Grant, and J. B. Lindsley, Tenn.

*On Medical Education*—E. Geddings, S. C.; C. W. LeBoutillier, Min.; G. F. Mitchell, O.; S. W. Clanton, Ala.; S. W. Butler, N. Y.

*Medical Literature*—R. Hills, Ohio, D. W. Yandell, Ky.; R. R. Porter, Del., H. A. Johnson, Ill.; C. E. Swan, Maine. Adopted.

To fill vacancies in the committee on Medical Topography and Epidemics—N. H. V. P. Fitch, Cal.; R. Murray.

To fill vacancies in the committee of Registration of Births, Marriages, and Deaths—A. T. Woodward, Vt.; R. W. Haxall, Va.; W. B. Casey, Conn.; A. R. Stout, Cal.

They recommended the continuance of the committee to procure Memorials of the eminent and worthy dead, and that the report, as far as prepared, be referred to the committee on Publication. Adopted.

The Secretary presented the report of Dr. W. H. Anderson, (absent) of Ala., on Medical Education, which was referred for publication.

Dr. Wroth sent through the Secretary so much of his report as was completed. His continuance was referred to the committee on Nomination, and the report accepted.

Dr. D. J. Cain, of S. C., presented his report upon Medical Topography and Epidemics, which was referred for publication.

Dr. E. D. Teriner, of N. O., also presented his report upon the same subject; which took the same course.

Dr. Gunn, from committee of Arrangements, asked that a special committee be appointed to whom several voluntary communications in hand should be referred. The articles referred to are as follow:

Dr. C. Q. Chandler, Wis., on Sulphate of Cinchona in periodic disease.

Isidor Gluck, on formation of gunshot wounds, etc.

G. P. Hackenburgh, on improved method of applying compression on the scrotum.

The committee to report on a uniform system of Registration of Births, Marriages, and Deaths, said they were unable to report in consequence of the death of their chairman, Dr. M. W. Wilson, of Conn.

Dr. Gross presented an invitation on behalf of the Medical Society of Louisville to this Association, to hold its meeting in that city in 1858. Placed on file.

Dr. Dorsey, of Ohio, offered the following:

*Resolved*, By the American Medical Association, that the committee on the Etiology and Pathology of Cholera, be instructed to memorialize the Congress of the United States, requesting that honorable body to grant every necessary assistance which can or will promote the objects for which the committee has been appointed. Carried.

Caspar Wister, of Pa., offered the following:

*Resolved*, That a committee of three be appointed by the President to correspond with the proper officers of the Smithsonian Institute, inquiring into the possibility of procuring a chamber in that institution for the use of this Association. Carried.

Dr. Wister, of Pa.; Hale, of D. C.; J. Neill, of Pa.; were appointed said committee.

Dr. Palmer, of Chicago, offered the following:

*Resolved*, That the volunteer communications in the hands of the committee of Arrangements be referred with other such communcations to a special committee, to be appointed by the Chair, at the place of publication of the transactions, and if in their judgment, the papers are worthy, they be referred by them to the committee on publication, to go into the transactions of the Association. Carried.

The Association then adjourned to Friday morning, at nine o'clock.

FOURTH DAY—MORNING SESSION.

The President called the Association to order. The Secretary read the minutes of the preceding session, which were corrected and approved.

Dr. Palmer of Ill., moved that Dr. Richard Coolidge, of D. C., be substituted in place of Dr. Finley, on the Committee to report on Epidemics. Carried.

Dr. Gunn, of Detroit, from Committee on Credentials, reported the names of new members, by invitation.

Dr. Atlee, of Pa. offered the following:

*Resolved*, That all voluntary communications hereafter presented to the Association, shall be referred to a special committee of ——, to be appointed by the President, on the first annual meeting, whose duty it shall be to examine such communications, and report upon the propriety of their presentation and reference to the Committee on Publications. Carried.

Dr. Palmer, of Chicago, moved that the above resolution, together with the suggestions in the report of the Committee on Prize Essays, as to whether any means can be devised to cause an increase of the number of Essays presented, be referred to a special committee, of which Dr. Leidy, of Pa., shall be chairman, to report to the next meeting of the Association. Carried. And G. B. Wood and C. D. Smith, of Philadelphia, were put upon said committee.

J. B. Lindsley, from the committee appointed to prepare a suitable minute, having reference to the death of P. C. Gooch, late Secretary of the Association, begged leave to report the following preamble and resolutions:

*Whereas*, the exhibition of high courage and self-sacrificing devotion to the good of others, is ever honorable to a profession by whose members it is manifested, and worthy of remembrance and emulation:

*Resolved*, That in the death of P. C. Gooch, of Richmond, Va., who nobly volunteered his services during the pestilence at Norfolk, we recognize a loss to this Association, the profession, and the country. His arduous and successful labors as Secretary of the meeting at Charleston and Richmond, merited the regard of this Association; the zeal, ability, and industry manifested by him as the founder and editor of the Stethoscope—the first medical periodical established in Va.—showed his devotion to the cause of medical progress and activity, and the manner of his death gave signal evidence that he was one of whom his country might well be proud.

*Resolved*, That a copy of these resolutions be transmitted by the Secretary to the relatives of the late Dr. Gooch. Carried.

Dr. Samuel Denton, of Michigan, offered the following:

*Resolved*, That a committee of three be appointed whose duty it shall be to enlist some enterprising publisher, and aid in collecting and arranging material for an American Medical Directory.

Laid on the table.

Dr. Smith, of N. Y., moved that a special committee be appointed to report at the next meeting of this Association, a classification of those diseases which involve a derangement of the mental manifestations. Carried; and leave given the mover to appoint the committee, he being chairman.

The Secretary read an invitation from Dr. Childs, of Boston, inviting the members of the Association to meet with the Massachusetts Medical Society on the first Wednesday of May. Accepted.

Dr. Atlee, of Pa., moved that a copy of the Association's transactions be sent to the Epidemological Society of London. Carried.

Dr. Gunn, of Mich., moved that any new Medical Society, not heretofore represented in this Association, be required to transmit to the Secretary, with the credentials of its delegates, the evidence of its existence, capacity, and good standing. Carried.

Dr. McGugan moved that a special committee be appointed to report on the subject of "Stomatitis Materna." Carried.

Dr. Bailey moved that Dr. N. S. Davis be requested to continue his observations on the subject of the changes produced in the composition and qualities of milk by pregnancy and menstruation; also, the best substitute for the mother's milk when weaning becomes necessary before the child is eighteen months old, and report at the next meeting of the Association. Carried.

Dr. Atlee, of Pa., moved that the thanks of the Association be tendered to all railroad companies who had furnished members with passes to this convention. Carried.

Dr. Palmer, of Ill., called for some disposition to be made of the resolutions appended to his report on the organization of Medical Societies.

On motion of Dr. Atlee, they were referred with the report for publication.

Dr. Palmer, of Ill., moved that the Association tender a vote of thanks to t e press of Detroit for the interest and attention given to their sessions in this city. Carried.

Dr. Palmer of Ill., moved that the committee on Registrations have leave now to present a partial report, which is hereby referred to the committee on publication. Carried.

Dr. Lindsley, of Tenn., offered the following:

*Whereas*, it is the object of this Association, in the award of its prizes for communications upon subjects appertaining to medical science, to encourage the progress of the latter, and as this result cannot be better attained than through original investigation and diseovery:

*Resolved*, That hereafter an annual prize of $100 be awarded for the best memoir or essay, founded on original investigation of the author, and in case of no memcir or essay being presented worthy of such award, the prize money to be appropriated towards the expense of publishing and illustrating such memoirs as may be subsequently deemed worthy of an award. Carried.

On motion, the Association then adjourned to meet at Nashville, Tenn., on the first Tuesday of May, 1857.

